# Completed Suicide Linked to the COVID-19 Pandemic by Using the Psychological Autopsy Method in Sibiu County, Romania: Case Series and Literature Review

**DOI:** 10.3390/healthcare10122377

**Published:** 2022-11-26

**Authors:** Roxana-Mihaela Crisan, Ciprian Ionuț Băcilă, Paul-Cătălin Toboltoc, Silviu Morar

**Affiliations:** 1 Doctoral Department, Faculty of Medicine, Lucian Blaga University of Sibiu, 550024 Sibiu, Romania; 2 Forensic Department, County Clinical Emergency Hospital of Sibiu, Corneliu Coposu Boulevard 2-4, 550245 Sibiu, Romania; 3 ‘Dr. Gheorghe Preda’ Clinical Psychiatry Hospital, 550082 Sibiu, Romania; 4 Dental Medicine and Nursing Department, Faculty of Medicine, Lucian Blaga University of Sibiu, 550024 Sibiu, Romania; 5 Department of Anatomical Pathology, County Clinical Emergency Hospital of Sibiu, Corneliu Coposu Boulevard 2-4, 550245 Sibiu, Romania; 6 Preclinical Department, Faculty of Medicine, Lucian Blaga University of Sibiu, 550024 Sibiu, Romania

**Keywords:** completed suicide, psychological autopsy, risk assessment, COVID-19 epidemiology, SARS-CoV-2, COVID-19 pandemic

## Abstract

The COVID-19 pandemic is associated with suicide, as some data suggests. Our study aims to investigate the emergence of eleven completed suicide cases suspected to be linked to the COVID-19 pandemic during the restrictive measures imposed by the Romanian government, and to identify the consequences of mental health, suicidal motivation, and behavioral changes. To this end, we analyzed the deceased’s medical records and applied the psychological autopsy method to the relatives/caregivers of the deceased for a suicidal investigation history, within conducted free-flow discussions. To highlight behavioral changes that occurred in the distant antecedents as well as immediately before the suicidal act, we used two sets of closed questions comprised of fifteen alarm signs, including depressive and/or anxiety symptoms. Our results showed that a deterioration of the mental status, especially concerning depressive and anxiety symptoms, was evident in people without or with pre-existing psychiatric pathology. The suicidal motivation proved to be complex including, in addition to the SARS-CoV-2 infection, social and economic consequences of the COVID-19 pandemic. We noted an intensification of the investigated alarm signs and even the emergence of new warning signs in the recent antecedents. Based on our findings, we reaffirmed the important role of the psychological autopsy method in suicide investigation, proving that it can detect the specific impact of the COVID-19 pandemic on people prone to suicide. This impact can be psycho-emotional, social, and/or economical, and thus we can state that the COVID-19 pandemic and its consequences can be, at least, a triggering factor that enhances completed suicide risk. Further studies are needed in this particular area because correlations between the COVID-19 pandemic and completed suicide do not appear to be accidental.

## 1. Introduction

Health systems and governments around the world have tried to minimize the negative effect of the COVID-19 pandemic by making decisions that radically changed people’s lives and generated substantial uncertainty; in this situation, information has been vital [1].

The daily worries about COVID-19 issues can have a negative impact on the psychological adjustment of a person, as they need to cope with stress related to various potentially dangerous conditions, with the likelihood of developing depression and/or anxiety symptoms [2]. A positive diagnosis of SARS-CoV-2 infection, isolation, quarantine, social distancing, and other socio-economic consequences generated by this pandemic can lead to mental health distress triggering psychological mediators such as sadness, worry, fear, anger, annoyance, frustration, guilt, helplessness, loneliness, nervousness and stigma [3]. Individuals may experience these psychological mediators during the COVID-19 pandemic with subsequent negative consequences on mental health which, in extreme cases, can lead to suicidal behavior, such as suicidal ideation, suicide attempts, and even completed suicide [3].

Mental health experts have alarmed the world regarding a potential increase in suicide rates, using phrases such as ‘a suicide tsunami’, ‘dual suicide and COVID-19 pandemic’ and ‘suicide mortality’ [1]. In reality however, the overview from most national and regional/local health statisticians around the world is that there was actually a slight decrease in overall suicide rates, while other data claimed that there was no substantial increase in suicide rates during the first year of the COVID-19 pandemic [1,4,5,6,7,8,9,10].

Data on the Romanian suicide rate before the COVID-19 pandemic reported a decrease in 2019 compared to previous years [11]. According to the data from the National Institute of Statistics (NIS), in Romania, the number of suicides has been decreasing in recent years, and the pandemic has not brought about a dramatic change in the total numbers. Thus, in 2020, there were 1734 suicides compared to 2236 in 2015, or 1978 in 2016. The change is minor compared to 2019, when 1744 suicides were reported. The first six months of 2021 did not bring a change in this trend [12]. In this context, although the number of completed suicides is decreasing, it is important to identify completed suicides where the COVID-19 pandemic has had a contributive role in completed suicides, by detecting the characteristics of vulnerable people, especially those with psychiatric diseases.

According to Global Health Data Exchange (GHDx) data, in 2019, the prevalence of mental disorders globally was 970 million people, of which 2.2 million people were from Romania. Mental disorders in Romania represented 11.89% of the population, of which depressive disorders accounted for 3.51% and anxiety disorders for 3.86%. These values are close to the Central European average and lower than the values recorded globally, but well below those recorded for Western Europe [13]. Thus, until completed suicide, some symptoms of depression and/or anxiety are likely to appear and develop.

However, there have been reports of individualized cases of suicide that appeared to be related to COVID-19 [3]. We reported in Sibiu County, Romania, three patients with SARS-CoV-2 infection who completed suicide, in which we noted that the SARS-CoV-2 infection, even without significant clinical severity, nevertheless played an important role in the occurrence of suicide by triggering psycho-emotional consequences that developed on a depressive background; we also highlighted the presence of several other associated risk factors [14]. Other cases in which suicide attempts and completed suicides were associated with the COVID-19 pandemic have been described; for example, in a male individual from Romania [15]. Possible suicidal motives in the context of the COVID-19 pandemic were also specified in completed suicides in other countries such as Bangladesh and India [3,16,17,18,19], and psychosocial stressors related to the COVID-19 pandemic were noticed also in attempted suicides performed by two young women of minority population with no prior psychiatric illnesses [19].

Despite the challenges posed by pandemics, the association between pandemics and suicide is poorly supported in the literature [16]. However, international organizations have estimated an increased risk of suicidal behavior associated with the COVID-19 pandemic, with long-term mental health consequences, explaining the need to implement effective preventive strategies supported by vigilance and international collaboration [20]; for this to happen, as much information as possible is needed.

Research in suicidology has been provided during the COVID-19 pandemic by performing complex forensic, psychiatric and psychological examinations. The imbalance of mental status has been investigated both in suicide attempts and completed suicides [21]. For the latter, the information obtained during the forensic investigation is of high accuracy given the fact that, according to Romanian legislation, the autopsy of suicide cases is mandatory, and also because of the possibility of obtaining data by using the psychological autopsy. This method consists of a retrospective reconstruction of the life of the deceased, with the aim of a better understanding of his death. Thus, in addition to the forensic and judicial investigation, the psychological autopsy method provides supplementary information, obtained by conducting an interview with the relatives or caregivers of the deceased [22,23].

Based on these data, we acknowledged the need to further investigate this possible correlation between the COVID-19 pandemic and completed suicide. We chose to use the psychological autopsy method to approach committed suicide, in order to investigate in-depth the complexity of this phenomenon.

## 2. Materials and Methods

Out of the eighty-seven cases of completed suicide autopsied within the Forensic Department of Sibiu County, Romania, recorded during the emergency and alert states of the COVID-19 pandemic imposed by the Romanian government (from March 2020 to March 2022), in eleven cases the deceased’s relatives and caregivers mentioned that the COVID-19 pandemic had a contributory role. Three of these cases, diagnosed with SARS-CoV-2 infection, had been already investigated by us within a pilot study, published in Applied Sciences in 2021 [11].

To identify all the psycho-emotional consequences that potentially can occur during the COVID-19 pandemic, including in patients that were not diagnosed with SARS-CoV-2 infection, we applied the method of the psychological autopsy to all the eleven cases in which the COVID-19 pandemic was mentioned as a possible trigger for mental imbalance and subsequent suicide. We conducted an extended analysis that included also the first three investigated cases, to have an overview of the total number and motives of completed suicide cases related to the COVID-19 pandemic situation in Sibiu County.

From the necropsy reports of the Forensic Department of Sibiu County, we extracted relevant data regarding the medical history of the deceased (medical records provided before the necropsy), the demographic coordinates, and the suicidal method. We also obtained information regarding the blood alcohol level, an analysis that was performed within our Toxicology Laboratory using gas chromatography–mass spectrometry (GC–MS).

Complementarily, within the psychological autopsy method, we addressed questions to the relatives or the caregivers of the deceased about the information they considered relevant to the reasons that led to suicide (including the suspected motivation for the suicidal act). We also investigated the psychiatric history (diagnoses, psychiatric admissions/consultations) of these deceased, and we gathered information relevant to suicide antecedents (prior suicide attempts, cases of suicide in the family). We also investigated the possible role played by the harmful consumption of alcohol in the emergence of suicide. At the same time, we focused on the possible role played by the COVID-19 pandemic in the deterioration of the mental state. To obtain extensive information in this regard, we conducted a free-flow discussion so that the relatives/caregivers could talk openly about the suffering that the suicidal person went through, which could have triggered self-harm. To highlight possible behavioral changes that occurred in the distant antecedents as well as immediately before the suicidal act, we used two sets of closed questions comprised of the same fifteen alarm signs/behavioral changes that were potentially noted in the distant antecedents as well as before death (signs that newly appeared or intensified). We chose the following 15 alarm signs: 1. communication of suicidal ideation (did he say he will ‘kill himself’?); 2. sadness; 3. tendency to isolation; 4. restlessness (as an indicator of anxiety); 5. aggression; 6. nervousness; 7. insomnia; 8. chronic fatigue; 9. lack of participation in family life; 10. lack of participation in social life; 11. feelings of worthlessness; 12. feelings of guilt (did he consider himself guilty for everything?); 13. feelings of inferiority; 14. lack of self-confidence; 15. loss of interest in life.

The respondents gave consent for using these data for scientific research, with the assurance of confidentiality of the research data according to the legislation in force regarding the protection of personal data. While applying the questionnaire, we took into account the particular ethical challenges of such an endeavor. An interview with a family member about such a delicate and controversial subject is not an easy thing to do. Trust, empathy, and confidentiality are essential elements. We were also careful to reduce as much as possible the psychological pressure that the respondents might experience. At the same time, we made it clear to them that the interview could stop at any time, should the sentiment of grief become unbearable.

The data obtained this way were processed using Microsoft Office Excel 2019.

## 3. Case Series Results

The eleven cases we selected for our analysis occurred as follows: one case was identified during the emergency state, and the rest of the cases during the alert state imposed by the Romanian government. We found a much higher prevalence of male gender (gender ratio M/F 4.5:1). Ten cases (nine men and one woman, ages 12–84 years) came from an urban environment; only one female, aged 41, lived in a rural area. SARS-CoV-2 infection (without significant clinical severity) was diagnosed before the suicidal act in five of the cases presented. Two of the deceased went through the COVID-19 disease a few months earlier. The last case described had an inconclusive COVID-19 test before his death. Five of the cases had no documented SARS-CoV-2 infection, but the respondents attributed a significant role to the COVID-19 pandemic in the emergence of the suicidal act. The preferred suicide method was hanging.

Following the investigation, we collected the data of suicide interest; this is presented by category in tabular form to highlight both the common characteristics and the particularities of each case.

### 3.1. General Data and Data Regarding Mental Status Obtained from the Analysis of Necropsy Reports and from Applying the Psychological Autopsy Method

Aspects Regarding Demographic Aspects, Suicide Method and Blood Alcohol Level—Obtained from the Necropsy Reports (Table 1)

Medical History (the Presence/Absence of SARS-CoV-2 Infection, Psychiatric and Somatic Diseases), Declared Alcohol Consumption, Prior Suicide Attempts, History of Suicide in the Family, the Place Where the Suicidal Act Occurred Are Presented in Table 2.

### 3.2. Suicidal Motives

Free dialogue with the respondents was aimed at revealing the suicidal motivation and as many details as possible on the suicidal person’s life. By using this psychological autopsy method, we highlighted the most stressful events in the deceased’s life (with emphasis on those that occurred during the COVID-19 pandemic), presented in chronological order in Table 3. On-site investigation revealed a farewell letter in three of these cases (cases no. 3, 6 and 11).

### 3.3. Behavioral Changes

The behavioral changes were assessed by investigating the emergence of one or more of the fifteen warning signs considered to foreshadow the suicidal act, as part of an under-diagnosed depressive syndrome. We compared the emergence of these alarm signs in the distant antecedents with those that appeared or intensified immediately prior to death. This analysis is detailed in Table 4.

## 4. Discussion

### 4.1. Forensic Considerations

The application of psychological autopsy in current practice within forensic methodology has played an important role in suicide investigation, and its contribution is undeniable [24,25]. It can assess the psychological framework of suicidal individuals for forensic purposes, especially when there is no suicide note [26,27,28]. Complementary to the necropsy findings that determine the manner of death and the method of suicide, it makes a substantial contribution to the positive diagnosis of suicide by highlighting the reasons and risk factors for suicide. By applying this method, we found that the relatives/caregivers of the deceased identified psycho-emotional consequences of the COVID-19 pandemic in 12.64% of the completed suicides in Sibiu County, Romania, that occurred during this pandemic. The literature data assert a possible connection between suicide and the consequences of the COVID-19 pandemic [3,14,15,16,17,18,19].

Research on completed suicide during the COVID-19 pandemic supports the understanding of suicidal behavior and its prevention during the pandemic. Ethical challenges of completed suicide include the assessment of risks (such as potential stigma) to family/caregivers [29]. The literature suggests the existence of emotional distress for interviewers and respondents as the most prominent burden [30]. For all the respondents, we ensured the safety, confidentiality, and integrity of all persons involved in the research area for suicide.

Suicide by hanging is the most common method of suicide in both sexes worldwide [31,32]. The hypothesis that suicide by hanging is the most frequently used method was also confirmed in our selected cases. However, we noted that the preferred method in controlled environments—which, in our case, is the hospital—was voluntary fall from a height. Three cases of suicide were carried out in the hospital; one case by hanging, and two cases by voluntary fall from height, all with SARS-CoV-2 infection. According to the literature data, only a small proportion (about 10%) of suicides occur in controlled environments (hospitals, prisons, police custody, etc.), and the rest occur in the community [32]. It is suggested that there is a tendency to resort to other methods of suicide in inpatients in medical settings relative to the usual suicide methods; also, those who use these non-specific suicide methods may have a different demographic profile [33].

We also documented a case of suicide of a 12-year-old girl, which was by hanging. The literature data show that hanging is the most common suicide method seen in children. Suicide among children deserves special attention because it has been suggested that suicide is the leading cause of death of young people in low- and middle-income countries, and the second leading cause in high-income countries [34,35].

A clear impact on mental health during COVID-19 was also described in a study in Cluj County, Romania, where the overall suicide rate remained the same while the number of suicides related to substance use disorders decreased [36].

### 4.2. Drinking Habits: Acute Alcohol Consumption (Detected in the Corpse’s Blood)

There is a link between alcohol consumption patterns and social, cultural, and health processes [37]. The outbreak of an epidemic (such as COVID-19) may induce changes in drinking behavior. Some authors even suggest that there is a subgroup of drinkers who are at risk of developing dangerous patterns of alcohol consumption during the lockdown [38]. Some data in the literature refer to increased consumption of alcohol due to isolation, or possibly because of rumors that alcohol might have a protective effect against the virus [37].

In our study, daily consumption of alcohol was not affirmed by the respondents; only one of the cases was known for alcohol use disorder, with declared weekly consumption. Examination of the alcohol level in the blood collected during the autopsy showed acute alcohol consumption in only two cases. A link between the COVID-19 pandemic, suicide, and alcohol consumption could not be documented in our cases. Nevertheless, the literature specifies a correlation between suicide and alcohol consumption. This is supported by the fact that alcohol increases impulsivity and self-aggression, and can promote depression and, ultimately, suicide in the context of the association with other relevant risk factors [38,39,40,41]. We believe that a potential link between alcohol, suicide, and the COVID-19 pandemic should be further explored.

### 4.3. Socio-Demographic Risk Factors

Males have always had higher completed suicide rates than females and epidemiological data are unanimous in confirming this fact [42,43]. The percentage of men who commit suicide is approximately 80% of all suicides [44]. We found an even higher prevalence of male gender (gender ratio M/F 4.5:1), so we suspect that men are at a higher risk to commit suicide in the context of the COVID-19 pandemic, as some reported cases suggest [3,18].

The age range of our cases was between 12 and 84 years, but we noted that eight cases were over 64 years old, retired, and from an urban environment. Consistent with our findings, the literature suggests that urban men over 65 years old are at the highest risk of suicide [45]; therefore, we consider it critical to further investigate the reasons and risk factors of suicide in this category [46,47], especially when we try to establish a connection with the COVID-19 pandemic.

According to our casuistry, suicide occurred in widowers, divorced, single, and even married people (a total of 8 cases in our study). We identified only three married people in our study, which raises the question of the possible role of an anomic marital status (single, divorced, widower) in making these individuals more vulnerable when confronting the consequences of the COVID-19 pandemic. We consider the literature data indicating that the family should play an important preventive role [48] to be correct (even though family tensions were surely to arise after the onset of the COVID-19 pandemic), but our findings show that these potential preventive measures would have less impact in a pandemic context.

The population of Sibiu County comprises predominantly Romanian Orthodox people but also has a minority of Hungarian and German people. Within our cases, we found 7 Orthodox Romanians but we also described a Hungarian Orthodox and a German Evangelical. In this context, we cannot attribute any particular role to the religious background with regards to the emergence of suicide.

### 4.4. Somatic and Neuropsychiatric Diseases

Seven of our cases had a confirmed positive test for SARS-CoV-2 infection. Five had the SARS-CoV-2 infection at the time of suicide, and one had an inconclusive COVID-19 test before the suicidal act. Neurotropism of this virus is known to affect the central and peripheral nervous systems [49,50,51,52,53,54]. It has been suggested that, in SARS-CoV-2 infection, neuropsychiatric symptoms occur in the acute phase through the onset of psychosis, insomnia, and general mood changes; but also after the infectious episode, through the onset of posttraumatic stress, panic attacks, and anxiety [55,56,57]. Even though SARS-CoV-2 infection may play a role in the increase of suicidal behavior, especially when associated with other multiple risk factors, our casuistry could not prove a causal link; further research is recommended in order to establish this association more precisely [57].

Only two of our cases (females) was not associated with any somatic or neuropsychological pathology in the past, but the psychological autopsy revealed alarm signs (such as sadness, tendency to isolation, restlessness, insomnia, chronic fatigue, lack of self-confidence, and loss of interest in life). One of these cases was the one of a 12-year-old; the lack of prior psychiatric disease in this particular case came as no surprise given the fact that lower rates of psychopathology are observed in suicide among children [34]. The literature data support the inclusion of colleges in psychological autopsy studies of suicide cases among children and adolescents as bringing added value, but further research is needed in this area, as well as additional information related to the impact of different events or even contagions on social networks [58]. An optimal approach is to collect information from other key informants [23] who may not be relatives or caregivers, but this was difficult in the pandemic context.

The most common neuropsychological finding was depression, the most significant suicide risk factor described in the literature [16,59,60,61]. Four of these cases were known to have diagnosed depression, and only one had a history of suicide attempts and suicide in his family.

The literature also suggests that other categories of individuals with a high-risk of suicide should not be neglected, such as women who may develop depression due to pregnancy, which may later lead to suicidal behavior. Several recent studies described a much higher prevalence of postpartum depression in the context of the COVID-19 pandemic, an increase that was also evident in Romania [62,63].

In Romania, the main factors related to suicide risk, even in the context of COVID-19, remained: previous suicide attempts, self-harm, and affective disturbances; anxiety and depression were detected as significant proximal predictors for suicide [64].

An impact on mental health during COVID-19 was also described in a study in Cluj County, Romania, where the overall suicide rate remained the same, but the number of suicides related to substance-use disorders decreased [36].

We also described the case of a 32-year old male known to have schizophrenia with recent psychiatric hospitalization. Suicides have been reported in patients with psychotic disorders on the schizophrenia spectrum, with estimates that a person who has had at least one psychotic experience over time is twice as likely to have suicidal ideation, three times as likely to attempt suicide, and four times as likely to die by suicide [65].

Within our cases, we found several that were known to have somatic diseases (recurrent ischemic strokes, chronic obstructive pulmonary disease, ischemic cardiomyopathy, prostate adenoma, prostate cancer, old spinal fracture), many of them with significant symptoms that severely impacted their quality of life (shortness of breath, chronic pain, chronic fatigue, urinary dysfunction). Almost all medical conditions in which physical health impairment was present are associated with an increased risk of suicide, which is substantially increased if several physical conditions are associated [66]. In addition, terminal medical illnesses are perceived as a burden that may trigger suicidal ideation resulting in completed suicide [65].

We consider that people with suicide risk factors are more vulnerable during a pandemic, with a greater need for mental health services. The COVID-19 pandemic has also had a negative impact on the accessibility of treatment (either psychiatric or somatic, or both), thus increasing suicide risk, as treatment continuity is crucial to prevent mental and physical deterioration in these patients [67,68]. Our last documented case is relevant in this regard because the inability of getting chemotherapy seems to have partly triggered the suicide act.

### 4.5. Suicidal Motives

All selected cases included (as selection criteria) the possible role of the consequences of the COVID-19 pandemic as a suicide risk factor. The manner in which the disease impacted the individual was complex: examples included the loss of a family member to COVID-19, the SARS-CoV-2 infection itself, the persistence of this disease longer than expected (over a month), fear of spreading the disease to family members, fear of being ‘trapped’ in the hospital, and difficulties in receiving proper cancer treatment in the context of an inconclusive COVID-19 test. The suicidal motivation proved to be complex, including, in addition to the SARS-CoV-2 infection, other social and economic consequences that appeared in the context of the COVID-19 pandemic: financial problems due to the pandemic situation, difficulties linked to new working conditions generated by the pandemic, and the lack of socialization (including quarantine and the need for online education) imposed by the COVID-19 pandemic.

However, we should keep in mind that the impact of the SARS-CoV-2 infection or the COVID-19 pandemic is significantly high in individuals that have had previously documented suicide-risk factors, such as somatic diseases with significant physical impairment, psychiatric diseases (especially depression and schizophrenia), loss of a family member (not COVID-19 related), other financial problems (pre-existing at the onset of the pandemic). Thus, we cannot state a causal connection between the consequences of the COVID-19 pandemic and the completed suicide act, but we can take into consideration their trigger role in the emergence of the suicidal act.

Furthermore, the literature data also suggests that all the changes that occur during the COVID-19 pandemic (illness due to SARS-CoV-2 infection, loss of a family member, stress caused by the hospital environment, deprivation of freedom, isolation, social restrictions, etc.) may trigger suicidal behavior; some authors even suggest that this pandemic can generate neuropsychiatric pathology, including major depressive disorder, bipolar disorder, various psychoses, and post-traumatic stress disorder [3,16,17,18,19]. Still unknown neuropsychiatric sequelae generated by this pandemic require ongoing research in this area, in order to implement appropriate prophylactic and therapeutic strategies in the long term. We believe that the long-term effects of the consequences of the pandemic need to be known and should not be undervalued, even once the pandemic is over.

### 4.6. Behavioral Changes

We found an evident deterioration of the mental status in the context of the COVID-19 pandemic (especially concerning depressive symptoms). This was seen both in people with pre-existing psychiatric pathology and in those without psychiatric antecedents, even though we could not document any psycho-emotional consequences due to the COVID-19 pandemic in most completed suicides (87.35%) that occurred in Sibiu County, Romania, during the COVID-19 pandemic.

**Figure 1:**
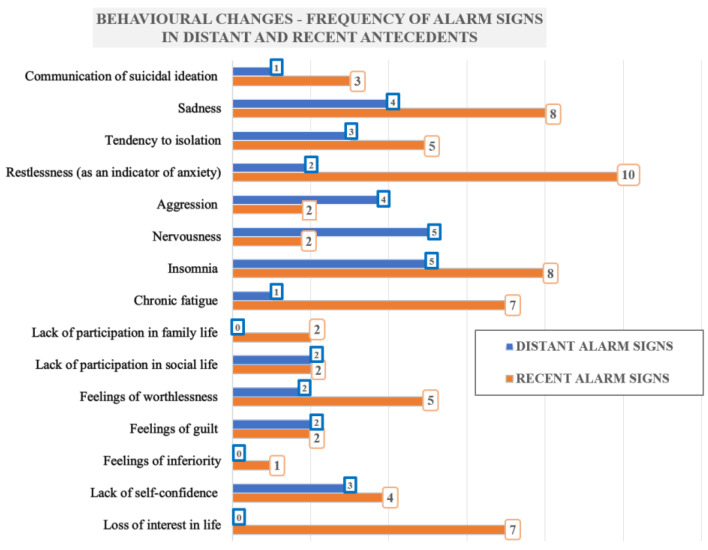
Variation of behavioral changes in the distant and recent antecedents.

The most frequent alarm signs, in descending order, in our selected cases were: restlessness (as an indicator of anxiety), sadness, insomnia, chronic fatigue, loss of interest in life, the tendency to isolation, and feelings of worthlessness. These are depicted in Figure 1. 

We also noted that most of the investigated alarm signs (with the notable exception of aggression and nervousness) were more frequently encountered in the period that preceded the completed suicide act. There was an increase in these, especially restlessness (as an indicator of anxiety), sadness, insomnia, chronic fatigue, loss of interest in life, and the feeling of worthlessness (Figure 1)—all classical indicators of a depressive state. Thus, we can state that, in most of these cases, the investigated symptoms intensified or new alarm signs appeared. As a prophylactic measure, clinicians should be aware that these changes can represent significant triggering factors for suicidal behavior, especially in patients with a history of SARS-CoV-2 infection and with other associated suicidal risk factors, even though these changes in suicidal behavior during the COVID-19 pandemic are not specific [38].

Out of the eleven cases, two showed none of the fifteen alarm signs in the distant antecedents but, in all cases, we documented one or more of these warning signs appearing or intensifying before the suicidal act. Alarm signs like sadness, restlessness, the tendency to isolation, insomnia and, in some cases, aggressiveness and nervousness are common and non-specific, but these were described in the literature as suicide risk factors during the COVID-19 pandemic [8,9,18,55,68,69,70,71,72,73,74].

Our comparative analysis of the suicide alarm signs in the distant antecedents versus the recent antecedents revealed an increased frequency and an intensification of these fifteen alarm signs, and even the emergence of new warning signs, in the context of the COVID-19 pandemic.

To summarize, our investigation focused on eleven cases of suicide that occurred in Sibiu County, Romania, during the emergency and alert states imposed by the Romanian government, in which a potential link to the COVID-19 pandemic was suspected. Suicide risk proved to be higher in males, aged 65+, from urban areas, with an anomic marital status, with or without pre-existing psychiatric pathology. The most common suicide method was hanging, but we also documented cases of voluntary falling from height (especially during hospitalization). Our comparative analysis of fifteen alarm signs (characteristic of a depressive disorder) in the distant antecedents versus recent antecedents revealed an increased frequency and an intensification of these fifteen alarm signs, and even the emergence of new warning signs in the context of the COVID-19 pandemic.

Highlighting these changes can be useful for early detection of the emergence of completed suicide in the post-COVID-19 era, especially taking into account the new economic challenges that some authors predicted would have an impact on the increase of suicidal behavior worldwide and in Romania [75].

## 5. Conclusions

Based on our findings, we highlight and reaffirm the important role of the psychological autopsy method in suicide investigation with regard to the detection of suicidal risk factors, motives, and behavioral changes. Moreover, we proved that this method can detect the specific impact of SARS-CoV-2 infection and the COVID-19 pandemic on people prone to suicide. Based on the investigated risk factors (gender, age, environment, marital status, organic and psychiatric disease), we conclude that the impact of the COVID-19 pandemic can be psycho-emotional, social, and/or economical; thus, we can state that the COVID-19 pandemic and its consequences can be a significant factor that triggers a completed suicide act. Our investigation also detected the presence of alarm signs (characteristic of a depressive disorder), an intensification of these alarm signs, and even the emergence of new warning signs in the context of the COVID-19 pandemic. Identifying these changes can be useful for the early detection of the emergence of completed suicide in the pandemic context.

The psycho-emotional impact of the COVID-19 pandemic may also have long-term consequences. Further studies are needed in this particular area because correlations between the COVID-19 pandemic and completed suicide do not appear to be accidental. These studies will have to identify the specific suicidal risk factors in the pandemic context because there is a need for prophylactic interventions in order to prevent committed suicide determined by the long-term consequences of the COVID-19 pandemic.

## Figures and Tables

**Table 1:** Gender, age, marital status, occupation, nationality, religion, suicide method and blood alcohol level.

Case	Gender	Age (Years)	Marital Status	Occupation	Nationality	Religion	Suicide Method	Blood Alcohol Level
1 *	M	77	single	retired	Hungarian	Orthodox	hanging	0
2	F	41	widow	unskilled worker	Romanian	Orthodox	hanging	0
3 *	M	73	married	retired	Romanian	Pentecostal	falling from a height	0
4 *	M	81	widower	retired	Romanian	Catholic	hanging	0.97
5	M	32	single	unemployed	Romanian	Orthodox	falling from a height	0
6	M	64	widower	retired	Romanian	Orthodox	hanging	0
7	F	12	single	pupil	Romanian	Orthodox	hanging	0
8	M	81	married	retired	German	Evangelical	hanging	0
9	M	64	divorced	retired	Romanian	Orthodox	falling from a height	0
10	M	74	married	retired	Romanian	Orthodox	hanging	0
11	M	84	divorced	retired	Romanian	Orthodox	hanging	1.99

* cases presented in the pilot study; M—male; F—female; blood alcohol level—g ‰ (grams per thousand mL).

**Table 2:** Medical history data, declared alcohol consumption, prior suicide attempts, history of suicide in the family and the place where the suicidal act occurred.

Case	SARS-CoV-2 Infection	Psychiatric Diseases	Psychiatric Admissions/Consultations	Somatic Diseases	Declared Alcohol Consumption	Prior Suicide Attempts	History of Suicide in the Family	The Place of the Suicidal Act
1 *	present	depression	absent	urinary tract infection; prostate adenoma	occasional	absent	absent	hospital—Infectious Diseases Department
2	absent	absent	absent	absent	not at all	absent	absent	home
3 *	present	absent	absent	chronic ischemic cardiomyopathy; prostate adenoma	not at all	absent	absent	hospital—Pneumology Department
4 *	present	depression	absent	chronic liver disease; atherosclerosis; anemia	occasional	absent	absent	home
5	absent	schizophrenia	recent admission and consultation	absent	occasional	absent	absent	at a dam
6	absent	depression	an old admission (10 years ago).	old spinal fracture with chronic pain; herniated disc	occasional	absent	absent	home
7	absent	absent	absent	absent	not at all	absent	absent	home
8	present	depression	recent consultation	chronic ischemic cardiomyopathy; prostate adenoma; recurrent ischemic strokes	not at all	by hanging and self-cutting	a sister-in-law (by hanging)	home
9	confirmed 4 months ago	alcohol use disorder	recent consult	chronic obstructive pulmonary disease	once a week	absent	absent	hospital—Pneumology Department
10	present	absent	absent	chronic ischemic cardiomyopathy; prostate adenoma	not at all	absent	absent	home
11	confirmed 2 months ago; with inconclusive COVID-19 test before the death	absent	absent	prostate cancer	occasional	absent	absent	home

* cases presented in the pilot study.

**Table 3:** Suicidal motives.

Case	Suicidal Motives
1 *	His diseases, including SARS-CoV-2 infection; The hospital quarantine he was subjected to due to SARS-CoV-2 infection during the pandemic emergency state (he did not like the idea that he was hospitalized in the ‘red zone’).
2	Her husband died eight months earlier; Financial problems (due to the COVID-19 pandemic—‘it was not so easy to find work’, ‘she had debts’).
3 *	He took care of his brother with SARS-CoV-2 infection, who unfortunately died; He got the infection from his brother; He was confirmed with SARS-CoV-2 infection by multiple positive tests (and was subsequently quarantined in hospital for about a month); He was concerned about the persistence of the SARS-CoV-2 infection (which went on for over a month); Fear of spreading the infection to family members; The relationship with his family deteriorated considerably (‘he no longer wanted to communicate’).
4 *	His wife died almost a year ago; His diseases, especially depression (he considered himself powerless due this illness); The home quarantine due to his SARS-CoV-2 infection imposed on him during the pandemic alert state; Fear of spreading the infection to family members; Loneliness caused by social distancing, especially with regard to family members (in the context of imposed home quarantine).
5	He suffered from schizophrenia (in remission under treatment); He expressed dissatisfaction regarding the fact that, due to the COVID-19 pandemic, his work schedule changed, involving a schedule with more hours of work (twelve hours of work followed by twenty-four hours off); he could not cope with the extended program and resigned; He later had difficulties finding another job due to his psychiatric medical history and the pandemic context; Deep down he considered that he would never be able to adapt to any new work conditions in the pandemic context.
6	His wife died almost eight months ago; He had financial problems; Chronic pain because of an old spinal cord fracture; He was afraid of the COVID-19 pandemic; he didn’t want to end ‘locked up’ in the hospital.
7	She was introverted; Lack of participation in social life in the pandemic context (before the COVID-19 pandemic she was socially active only at school); school was online during the alert state of the COVID-19 pandemic, thus she lacked contact with her friends; The family could not provide another reason to explain her suicide.
8	He previously was hospitalized for two strokes, and was recently hospitalized with SARS-CoV-2 infection. Each time after a short period he refused to be hospitalized; he signed and assumed all the consequences of hospital discharge just to be able to leave the hospital; He apparently became more and more ‘stressed’ about a potential future hospitalization; Before the suicidal act he stated that ‘something was wrong with him’.
9	Chronic obstructive pulmonary disease (with oxygen requirement); Diagnosed with SARS-CoV-2 infection four months ago; Recently he was also suspected to have lung cancer; His relatives suspected that his lung affliction since he contacted the SARS-CoV-2 infection might have triggered the suicidal act.
10	Prostate adenoma with significant urinary discomfort; His wife was picked up by ambulance and hospitalized for SARS-CoV-2 infection the very day he committed suicide; Even though he was also diagnosed with SARS-CoV-2 infection, his general status was good, with minimal symptoms, thus he refused hospitalization and medical investigations.
11	Confirmed SARS-CoV-2 infection about three months ago; His son was diagnosed with SARS-CoV-2 infection (with minimal symptoms) at the time of his suicidal act; Due to his prostate cancer, he was ‘in great pain’; He was disturbed that, due to an inconclusive COVID-19 test, his ongoing chemotherapy had to be postponed, and his pain was not improving.

* cases presented in the pilot study.

**Table 4:** Behavioral changes.

Case	Alarm Signs in the Distant Antecedents	Alarm Signs in the Close Antecedents
1 *	sadness, restlessness, nervousness	sadness, tendency to isolation, restlessness, aggression, nervousness, insomnia, chronic fatigue.
2	insomnia, chronic fatigue, lack of self-confidence	restlessness, insomnia, chronic fatigue, lack of self-confidence, loss of interest in life
3 *	sadness, tendency to isolation, aggression, nervousness, feelings of guilt	sadness, tendency to isolation, restlessness, aggression, nervousness, insomnia, chronic fatigue, lack of participation in social and family life, feelings of guilt
4 *	aggression, nervousness, feelings of worthlessness, feelings of guilt, lack of self-confidence	communication of suicidal ideation, sadness, tendency to isolation, restlessness, insomnia, chronic fatigue, lack of participation in social and family life, feelings of worthlessness, feelings of guilt, lack of self-confidence, loss of interest in life
5	sadness, tendency to isolation, lack of participation in social life, lack of self-confidence	restlessness, feelings of worthlessness, feelings of inferiority, lack of self-confidence, loss of interest in life
6	sadness, restlessness, insomnia	communication of suicidal ideation, sadness, tendency to isolation; restlessness, insomnia; chronic fatigue, feelings of worthlessness, lack of self-confidence, loss of interest in life
7	tendency to isolation, insomnia	sadness, tendency to isolation, insomnia
8	communication of suicidal ideation, aggression, nervousness, insomnia, lack of participation in social life, feelings of worthlessness,	communication of suicidal ideation, sadness, restlessness, insomnia, chronic fatigue, feelings of worthlessness, loss of interest in life
9	aggression, nervousness, insomnia	restlessness, insomnia, chronic fatigue, feelings of worthlessness.
10	none of the fifteen signs were detected	sadness, restlessness,
11	none of the fifteen signs were detected	sadness, restlessness, loss of interest in life.

* cases presented in the pilot study.

## Data Availability

The data presented in this study are available on request from the corresponding author. The data are not publicly available due to data-protection legislation.
